# Genome-wide QTL mapping for agronomic traits in the winter wheat cultivar Pindong 34 based on 90K SNP array

**DOI:** 10.3389/fpls.2024.1369440

**Published:** 2024-04-04

**Authors:** Liangqi Zhang, Yuqi Luo, Xiao Zhong, Guoyun Jia, Hao Chen, Yuqi Wang, Jianian Zhou, Chunhua Ma, Xin Li, Kebing Huang, Suizhuang Yang, Jianfeng Wang, Dejun Han, Yong Ren, Lin Cai, Xinli Zhou

**Affiliations:** ^1^ Wheat Research Institute, School of Life Sciences and Engineering, Southwest University of Science and Technology, Mianyang, Sichuan, China; ^2^ Chongqing Banan District Agricultural Technology Promoting Station, Chongqing, China; ^3^ State Key Laboratory of Crop Stress Biology for Arid Areas, College of Plant Protection, Northwest A&F University, Yangling, Shanxi, China; ^4^ Crop Characteristic Resources Creation and Utilization Key Laboratory of Sichuan Province, Mianyang Institute of Agricultural Science, Mianyang, Sichuan, China; ^5^ College of Tobacco Science of Guizhou University, Key Laboratory of Plant Resource Conservation and Germplasm Innovation in Mountainous Region (Ministry of Education), Guizhou Key Lab of Agro-Bioengineering, Guiyang, China

**Keywords:** wheat, agronomic traits, wheat 90K SNP array, genetic map, QTL

## Abstract

**Introduction:**

Agronomic traits are key components of wheat yield. Exploitation of the major underlying quantitative trait loci (QTLs) can improve the yield potential in wheat breeding.

**Methods:**

In this study, we constructed a recombinant inbred line (RIL) population from Mingxian 169 (MX169) and Pindong 34 (PD34) to determine the QTLs for grain length (GL), grain width (GW), grain length-to-width ratio (LWR), plant height (PH), spike length (SL), grain number per spike (GNS), and the thousand grain weight (TGW) across four environments using wheat 90K SNP array.

**Results:**

A QTL associated with TGW, i.e., *QTGWpd.swust-6BS*, was identified on chromosome 6B, which explained approximately 14.1%–16.2% of the phenotypic variation. In addition, eight QTLs associated with GL were detected across six chromosomes in four different test environments. These were *QGLpd.swust-1BL*, *QGLpd.swust-2BL*, *QGLpd.swust-3BL.1*, *QGLpd.swust-3BL.2*, *QGLpd.swust-5DL*, *QGLpd.swust-6AL*, *QGLpd.swust-6DL.1*, and *QGLpd.swust-6DL.2*. They accounted for 9.0%–21.3% of the phenotypic variation. Two QTLs, namely, *QGWpd.swust-3BS* and *QGWpd.swust-6DL*, were detected for GW on chromosomes 3B and 6D, respectively. These QTLs explained 12.8%–14.6% and 10.8%–15.2% of the phenotypic variation, respectively. In addition, two QTLs, i.e., *QLWRpd.swust-7AS.1* and *QLWRpd.swust-7AS.2*, were detected on chromosome 7A for the grain LWR, which explained 10.9%–11.6% and 11.6%–11.2% of the phenotypic variation, respectively. Another QTL, named *QGNSpd-swust-6DS*, was discovered on chromosome 6D, which determines the GNS and which accounted for 11.4%–13.8% of the phenotypic variation. Furthermore, five QTLs associated with PH were mapped on chromosomes 2D, 3A, 5A, 6B, and 7B. These QTLs were *QPHpd.swust-2DL*, *QPHpd.swust-3AL*, *QPHpd.swust-5AL*, *QPHpd.swust-6BL*, and *QPHpd.swust-7BS*, which accounted for 11.3%–19.3% of the phenotypic variation. Lastly, a QTL named *QSLpd.swust-3AL*, conferring SL, was detected on chromosome 3A and explained 16.1%–17.6% of the phenotypic variation. All of these QTLs were defined within the physical interval of the Chinese spring reference genome.

**Discussion:**

The findings of this study have significant implications for the development of fine genetic maps, for genomic breeding, and for marker-assisted selection to enhance wheat grain yield.

## 1 Introduction

Wheat is one of the most important food crops in the world, producing approximately 700 million tons of wheat annually and providing nearly 20% of the energy source for the global population (https://www.fao.org/faostat/en) (Shewry, 2009; Juliana et al., 2019). With the continuous growth of the global population and the improvement of living standards, the demand for wheat is gradually increasing. However, the current yield level of wheat cannot meet the projected future demand. Wheat production will need to increase by almost 70% by 2050 in order to maintain food security and accommodate the heightened demand resulting from the expanding global population (Ray et al., 2013). Wheat yield is mainly determined by the grain number per spike (GNS), the spike number, and the thousand grain weight (TGW) (Cristina et al., 2021). Furthermore, various agronomic traits exert influence on wheat yield. Previous studies have indicated that plant height (PH) can impact the dry matter accumulation and lodging resistance, while panicle traits including the spike length (SL) and GNS, among others, and the grain characteristics such as grain length (GL), grain width (GW), and TGW, among others, exhibit positive correlations with yield (Elouafi and Nachit, 2004; Griffiths et al., 2012; Simmonds et al., 2014). Therefore, it is of great significance to understand the genes or quantitative trait loci (QTLs) associated with the agronomic traits related to wheat yield composition in wheat breeding (Yan et al., 2019).

Identification of the QTLs associated with important traits is one of the first steps in the development of wheat cultivars using molecular marker-assisted selection (MAS) (Collard and Mackill, 2007). The grain traits mainly include the TGW, GL, GW, and the grain length-to-width ratio (LWR), among others. Of these, TGW is a composite trait, while GL, GW, and LWR are its constituent elements (Campbell et al., 1999). The level of TGW in wheat is not only directly related to yield but also affects the flour milling and processing quality of wheat, as well as the vigor of wheat seedlings (Wiersma et al., 2001; Breseghello and Sorrells, 2006). Therefore, identification of the QTLs important for agronomical traits, such as PH and TGW, provides critical information beneficial to food security (Xiao et al., 2022). To date, 25 *Rht* genes (*Rht1–Rht25*) have been identified in wheat, with *Rht-B1*, *Rht-D1*, and *Rht8* being widely utilized dwarfing genes in contemporary breeding programs (Hedden, 2003; Chai et al., 2022). Dwarf or semi-dwarf varieties, compared with the tall phenotypes, exhibit enhanced allocation of photoassimilates toward spike development, leading to alterations in the spike morphology and to increased GNS. Previous studies have identified several QTLs associated with SL and GW (Sun et al., 2009; Williams et al., 2013; Tyagi et al., 2015; Kumar et al., 2016). However, studies on QTLs related to grain LWR remain limited (Li et al., 2015; Kumar et al., 2016; Kumari et al., 2018).

In the 1990s, the first genetic map of wheat was successfully constructed using restriction fragment length polymorphism (RFLP) markers (Devos et al., 1993). Subsequently, molecular markers such as random amplified polymorphic DNA (RAPD), amplified fragment length polymorphism (AFLP), and simple sequence repeat (SSR), based on PCR amplification technology, have emerged as the primary tools for genetic mapping. However, these markers exhibited limited genetic diversity, and the process of marker development was time-consuming and labor-intensive. Furthermore, the resulting maps lacked the required density for precise gene localization and cloning. Single nucleotide polymorphisms (SNPs) represent the most abundant and ubiquitous form of genetic variation within genomes (Kwok, 2001). SNPs offer superior value over other markers for high-density genetic mapping, fine-mapping of target genes, and gene cloning efforts (Rafalski, 2002). In recent decades, there has been significant interest in SNP research and development in both animals and plants, including wheat. Substantial progress has been achieved in the development and application of SNP markers in wheat. Genome-wide association studies (GWAS) have been successfully employed for polymorphism detection in both tetraploid and hexaploid wheat (Juliana et al., 2018). Currently, various SNP arrays, such as the 35K, 55K, 90K, and 660K SNP arrays, have been developed for wheat and utilized in QTL mapping experiments and in GWAS. For example, Zhou et al. utilized the 90K SNP array to map the loci associated with adult plant resistance (APR) to stripe rust in the common wheat cultivar Pindong 34 and the durum wheat Svevo (Zhou et al., 2021, 2022).

Pindong 34 (PD34) is a winter wheat cultivar bred in 1990 by the Institute of Crop Variety Resources, Chinese Academy of Agricultural Sciences. It exhibits a high level of resistance to stripe rust and most other wheat diseases and insect pests (Zhou et al., 2022). A total of 12 QTLs for APR, as well as three QTLs for all-stage resistance (including *Yr61*), were identified in PD34 using the 90K SNP-Chip array in the recombinant inbred line (RIL) population consisting of 119 lines (Zhou et al., 2022). Identification of the QTLs influencing grain yield and its related traits is necessary to more precisely define their inheritance. The objectives of this study were: 1) to dissect the QTLs affecting grain yield in the RIL population from a cross between Mingxian 169 (MX169) and PD34; 2) to determine the chromosome locations and phenotypic effects of these yield-related QTLs across environments and identify the molecular markers associated with these traits; and 3) to determine the physical position of each QTL in the Chinese spring (CS) reference genome (IWGSC Ref Seq v1.0; https://plants.ensembl.org/Triticum_aestivum/Info/Index) and compare these with the reported QTLs.

## 2 Materials and methods

### 2.1 Plant materials

The mapping population used in this study was derived from a cross of MX169 and PD34. It was advanced to the F_6:10_ generation using the single seed descent method and included 119 RILs (Zhou et al., 2022). PD34 was selected from the cross [(Yan7578/81/128)//176(15)9-26/Dongda 2], which has the characteristics of high TGW, large grains, and low PH (80 cm) (Zhou et al., 2014). MX169 is an ancient Chinese winter wheat variety with a high PH, a shorter GL, and a narrower GW than the PD34 variety.

### 2.2 Field experiments and investigation of agronomic traits

The experiment was conducted in four different environments. Phenotypic evaluations of the agronomic traits of the RILs and the two parents were conducted in the field at Yangling, Shanxi Province (34°17′ N, 108°04′ E, 519 m altitude), and Mianyang, Sichuan Province (31°33′ N, 104°55′ E, 485 m altitude), over two successive years (2019 and 2020). The experiment was conducted in a randomized block design with two replicates. The plots were 1 m in length and 30 cm apart. Approximately 20–30 seeds were planted in a row. At maturity, half-row plants were randomly selected from each line for the measurements of PH, SL, and GNS. PH was measured at harvest maturity from the ground level to the tip of the spike, excluding awns, using a 100-cm ruler. SL was measured from the base to the tip, excluding awns, using a 15-cm ruler. GNS measured the total number of grains on the spike of wheat determined from the mean of five random spikes. After manual threshing, the grain was air-dried to measure the grain traits. The GL, GW, TGW, and LWR were determined from the mean of five random spikes in a line using the Wanshen SC-G automatic seed test analyzer. The specific measurement results are shown in **Table 1**. Field management was the same as commonly practiced in wheat production, and all agronomic trait data were obtained under conditions without disease control.

**Table 1:** Statistical analysis for the phenotype data of the agronomic traits in the Mingxian 169 (MX169) × Pindong 34 (PD34) recombinant inbred line (RIL) populations from 2019 to 2020.

Trait	Environment	PD34	MX169	RIL min.	RIL max.	RIL avg.	*p*-value	*h* ^2^ (%)
GL (mm)	MY19	8.0	5.7	5.4	7.9	6.4	<0.01	0.82
	YL19	7.5	5.8	5.4	7.7	6.5	<0.01	
	MY20	8.0	5.7	5.3	7.7	6.4	<0.01	
	YL20	7.4	5.7	5.3	7.6	6.4	<0.01	
GW (mm)	MY19	2.9	2.3	2.0	4.1	2.6	<0.01	0.75
	YL19	2.6	2.8	2.3	6.0	2.9	<0.01	
	MY20	2.9	2.3	1.9	3.5	2.6	<0.01	
	YL20	2.5	2.7	2.2	3.5	2.9	<0.01	
LWR	MY19	2.8	2.5	1.6	3.2	2.5	<0.01	0.82
	YL19	2.9	2.1	1.1	3.1	2.3	<0.01	
	MY20	2.8	2.4	2.0	3.3	2.5	<0.01	
	YL20	2.9	2.1	1.7	3.0	2.3	<0.01	
PH (cm)	MY19	80.0	96.0	63.0	128.0	92.9	<0.01	0.76
	YL19	85.0	130.0	65.0	135.0	106.9	<0.01	
	MY20	93.0	92.0	77.0	140.0	110.3	<0.01	
	YL20	85.0	120.0	60.0	135.0	103.2	<0.01	
SL (cm)	MY19	11.6	6.3	5.7	15.1	9.4	<0.01	0.86
	YL19	12.7	6.4	4.2	12.6	8.0	<0.01	
	MY20	11.6	6.2	5.6	27.3	9.6	<0.01	
	YL20	12.7	6.2	4.2	12.9	9.0	<0.01	
GNS	MY19	51.0	38.0	13.0	71.0	38.0	<0.01	0.86
	YL19	55.0	36.0	11.0	86.0	39.0	<0.01	
	MY20	50.0	37.0	12.0	72.0	38.0	<0.01	
	YL20	56.0	36.0	11.0	84.0	39.0	<0.01	
TGW (g)	MY19	30.2	16.2	11.5	48.0	27.4	<0.01	0.80
	YL19	37.0	14.8	10.6	52.8	30.9	<0.01	
	MY20	35.2	15.5	7.1	48.2	24.1	<0.01	
	YL20	32.4	25.8	7.9	45.1	29.0	<0.01	

The broad-sense heritability, *h* ^2^, was used to compare the relationship between the magnitude of the effects of genetic and environmental factors on the phenotypic variation for a given trait. Heritability, *h* ^2^, ranges from 0 to 1, with 0 indicating that the phenotypic variation was due exclusively to environmental influences and 1 indicating that the phenotypic variation was due exclusively to genetic factors.

GL, grain length; GW, grain width; LWR, grain length-to-width ratio; PH, plant height; SL, spike length; GNS, grain number per spike; TGW, thousand grain weight.

### 2.3 Statistical analysis

The average phenotypic values of each trait in each line from both sites were used to calculate the variance of each trait in individual environmental analysis. IciMapping v4.1 (http://www.isbreeding.net/) was used in the software analysis of variance function to analyze variance (ANOVA) in the phenotype data and to calculate the generalized heritability. The broad-sense heritability ( $h^{2}$ ) of the phenotypic traits was estimated using the formula $\;h^{2}=\sigma_{\text{g}}^{2}/\left(\sigma_{\text{g}}^{2}+\sigma_{\text{ge}}^{2}/r+\sigma_{\varepsilon }^{2}/re\right)$ , where $\sigma_{\text{g}}^{2}$ is the genotype (line) variance; $\sigma_{\text{ge}}^{2}$ is the genotype × environment interaction variance; $\sigma_{\varepsilon }^{2}$ is the residual error variance; *e* is the number of replicates; and *r* is the number of environments. Pearson’s correlations between traits were calculated using SPSS.20 (https://www.ibm.com/cn-zh/spss).

### 2.4 Linkage map construction and QTL analysis

The mapping population comprised genetic data from 119 RILs genotyped using a 90K SNP-Chip genetic map, as reported by Zhou et al. (2022). The genetic map comprised a total of 11,346 markers, which were subsequently reduced to only 4,200 markers across 21 chromosomes by utilizing the “BIN” function in IciMapping v4.1 (Meng et al., 2015). The map had a genetic distance of 18,228.5 cM, with an average genetic distance of 868.0 cM. The GL, GW, LWR, PH, SL, GNS, and TGW data in each environment were used for QTL analysis with walking speed of 1 cM, value *p* for input variables (PIN) of 0.0001, logarithm of odds (LOD) of 2.5, and a window size of 5 cM. Moreover, possible QTLs were detected. When the LOD value was greater than 2.5, a QTL was identified explaining the phenotypic variation and an additive effect value for each QTL was calculated.

## 3 Results

### 3.1 Phenotypic variation and correlation analysis

**Figure 1:**
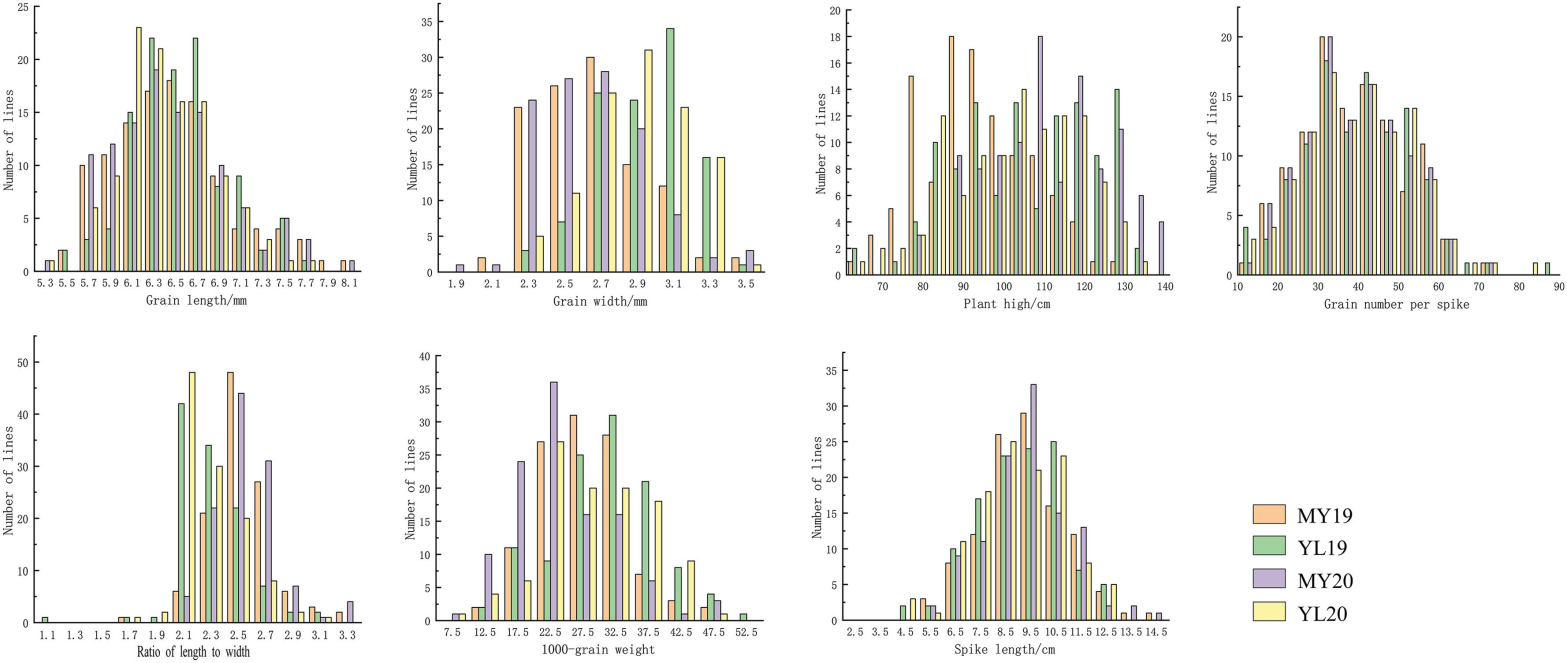
Frequency distributions of the mean grain length, grain width, grain length-to-width ratio, thousand grain weight, plant height, grain number per spike, and spike length, for 119 recombinant inbred lines (RILs) from the cross Mingxian 169 (MX) × Pindong 34 (PD) grown in Mianyang (MY) and Yanglin (YL) in 2019–2020.

In four different environments, PD34 exhibited a GL ranging from 7.4 to 8.0 mm, whereas MX169 displayed a GL range of 5.7–5.8 mm. The RIL population demonstrated GL variations from 5.4 to 7.7 mm, with mean values ranging from 6.4 to 6.5 mm across environments. For GW, that of PD34 ranged from 2.5 to 2.9 mm, while that of MX169 ranged from 2.3 to 2.8 mm. The RIL population displayed GW variations from 2.1 to 4.3 mm, with mean values ranging from 2.6 to 2.9 mm. The LWR for PD34 ranged from 2.8 to 2.9, whereas that for MX169 ranged from 2.1 to 2.5. The RIL population exhibited LWR variations from 1.6 to 3.2, with mean values ranging from 2.3 to 2.5. The PH of PD34 ranged from 80.0 to 93.0 cm, while that for MX169 ranged from 92.0 to 130.0 cm. The RIL population displayed PH variations from 66.3 to 134.5 cm, with mean values ranging from 92.9 to 110.3 cm across environments. The SL range for PD34 was 11.6–12.7 cm compared with 6.2–6.4 cm for MX169. The RIL population exhibited PH variations from 4.9 to 17.0 cm, with an average of 9.0–9.6 cm across environments. For GNS, that of PD34 ranged from 50.0 to 56.0, while that of MX169 ranged from 36.0 to 38.0. The RIL population displayed GNS variations from 11.0 to 78.0, with an average of 38.0–39.0 across environments. Regarding TGW, the range for PD34 was from 30.2 to 37.0 g, while that for MX169 was from 14.8 to 25.8 g. The RIL population demonstrated TGW variations from 9.3 to 48.5 g, with an average of 24.1–30.9 g (**Table 1**). According to the phenotypic distribution of the parental and RIL populations, the GL, GW, LWR, PH, SL, GNS, and TGW were all related to environmental conditions (**Figure 1**; **Table 1**). The GL, GW, LWR, PH, SL, GNS, and TGW all had high $h^{2}$ (75%–86%), indicating that all of the traits were stable and mainly determined by genetic factors. All traits exhibited transgressive segregation in both directions beyond the parental values, indicating quantitative traits under polygenic control, thereby rendering them suitable for QTL analysis (**Table 1**).

### 3.2 QTL analysis

**Figure 2:**
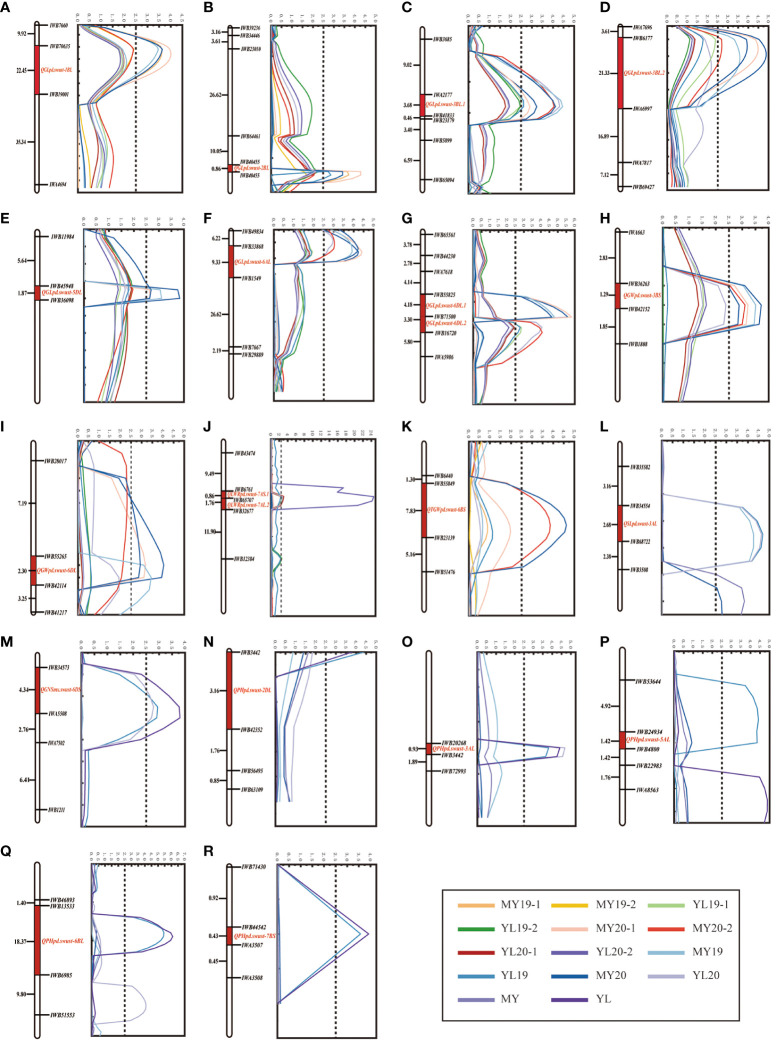
**(A–R)** Agronomic traits of the quantitative trait loci (QTLs) on the genetic map of chromosomes 1B, 2B, 2D, 3A, 3B, 5A, 5D, 6A, 6B, 6D, 7A, and 7B based on the agronomic data. The *y*-axis is in centimorgan (cM) distance and the *x*-axis indicates the logarithm of odds (LOD) scores. All LOD scores of the QTLs shown are similar. **(A–G)** Grain length (GL). **(H, I)** Grain width (GW). **(J)** Grain length-to-width ratio (LWR). **(K)** Thousand grain weight (TGW). **(L)** Spike length (SL). **(M)** Grain number per spike (GNS). **(M–R)** Plant height (PH).

The phenotypic of the GL, GW, LWR, PH, SL, GNS, and TGW data of the 119 RILs from the four field experiments were used for the identification of QTLs. Inclusive composite interval mapping (ICIM) identified a total of 20 QTLs (one for TGW, eight for GL, two for GW, two for LWR, five for PH, one for SL, and one for GNS) distributed on 12 chromosomes (1B, 2B, 2D, 3A, 3B, 5A, 5D, 6A, 6B, 6D, 7A, and 7B) within 18 genomic regions (**Figure 2**; **Table 2**). The additive effects of these QTLs on the GL, GW, LWR, TGW, and SL were all derived from PD34, and one QTL for the regulation of GNS was located on chromosome 6DS from MX169.

**Table 2:** Summary of the 20 quantitative trait loci (QTLs) identified on the agronomic characteristics of the Mingxian 169/Pindong 34 recombinant inbred line (RIL) populations examined in Yangling and Mianyang in 2019–2020.

QTL and trait	QTL	Environment	Marker interval	LOD	PVE (%)	Add
GL	*QGLpd.swust-1BL*	MY19	*IWB19001* *IWB70635*	3.5	13.5	0.21
		YL19		3.7	13.8	0.23
		MY20		3.6	14.0	0.21
		YL20		4.1	14.9	0.25
	*QGLpd.swust-2BL*	MY19	*IWB40455* *IWB61115*	3.1	11.7	0.19
		YL19		4.2	15.8	0.22
		MY20		3.7	13.9	0.21
		YL20		4.9	18.1	0.24
	*QGLpd.swust-3BL.1*	MY19	*IWB41833* *IWA2177*	4.5	16.0	0.23
		YL19		2.6	9.9	0.15
		MY20		4.1	14.8	0.22
		YL20		4.1	14.6	0.25
	*QGLpd.swust-3BL.2*	MY19	*IWB7177* *IWA6997*	4.1	15.9	0.22
		YL19		3.9	14.6	0.17
		MY20		4.6	17.6	0.23
		YL20		3.1	12.1	0.15
	*QGLpd.swust-5DL*	MY19	*IWB45948* *IWB36098*	3.1	11.4	0.19
		YL19		3.8	14.0	0.21
		MY20		2.7	10.0	0.18
		YL20		2.6	10.0	0.18
	*QGLpd.swust-6AL*	MY19	*IWB1549* *IWB33868*	4.4	15.2	0.24
		YL19		4.8	16.3	0.25
		MY20		4.6	15.9	0.24
		YL20		5.1	17.1	0.26
	*QGLpd.swust-6DL.1*	MY19	*IWB55825* *IWB71500*	4.8	17.7	0.24
		YL19		5.6	20.6	0.25
		MY20		4.6	17.2	0.23
		YL20		5.8	21.3	0.26
	*QGLpd.swust-6DL.2*	MY19	*IWB63232* *IWB16720*	3.7	14.2	0.26
		YL19		2.8	10.3	0.17
		MY20		3.5	14.2	0.23
		YL20		2.5	9.0	0.16
GW	*QGWpd.swust-3BS*	MY19	*IWB36263* *IWB42152*	3.5	13.5	0.12
		MY20		3.7	14.6	0.11
		YL20		3.2	12.8	0.11
	*QGWpd.swust-6DL*	MY19	*IWB42114* *IWB55265*	3.3	13.5	0.12
		YL20		4.0	15.2	0.13
		MY20		2.8	10.8	0.11
LWR	*QLWRpd.swust-7AS.1*	YL19	*IWB32677* *IWB65707*	2.7	11.6	0.08
		YL20		24.5	10.9	0.28
	*QLWRpd.swust-7AL.2*	MY20	*IWB65707* *IWB6761*	2.9	11.6	0.08
		YL20		2.8	11.2	0.07
TGW	*QTGWpd.swust-6BS*	MY19	*IWB55849* *IWB23139*	3.8	14.1	3.91
		MY20		4.6	16.2	3.57
SL	*QSLpd.swust-3AL*	MY19	*IWB34554* *IWB68722*	4.3	16.1	0.73
		YL19		4.7	17.6	0.71
		YL20		4.6	17.3	0.72
GNS	*QGNSmx.swust-6DS*	YL19	*IWB34573* *IWA5508*	2.9	13.8	−0.61
		YL20		2.8	11.4	−0.61
PH	*QPHpd.swust-2DL*	YL19	*IWB42352* *IWB3442*	4.4	17.4	−7.07
		YL20		3.8	14.8	−6.21
	*QPHpd.swust-3AL*	YL19	*IWB20268* *IWB52753*	3.8	15.2	−6.66
		MY20		4.4	17.3	−6.71
		YL20		4.7	19.3	−7.40
	*QPHpd.swust-5AL*	YL19	*IWB24934* *IWB4800*	4.7	17.2	−7.13
		YL20		3.0	11.3	−6.35
	*QPHpd.swust-6BL*	YL19	*IWB6985* *IWB13533*	3.5	13.4	−6.23
		MY20		4.6	16.9	−6.71
		YL20		4.2	15.9	−6.99
	*QPHpd.swust-7BS*	YL19	*IWB44542* *IWA3507*	3.6	14.1	−6.23
		YL20		3.9	15.0	−6.20

LOD, logarithm of odds score; PVE, percentage of the phenotypic variance explained by the individual QTLs; Add, additive effect of the grain yield allele; MY, Mianyang; YL, Yangling; GL, grain length; GW, grain width; LWR, grain length-to-width ratio; PH, plant height; SL, spike length; GNS, grain number per spike; TGW, thousand grain weight.

### 3.3 QTL for GL

There were eight QTLs detected for GL, and all eight were identified across all four environments, all of which originated exclusively from the parent PD34 (**Figure 2**). These stable QTLs were distributed on chromosomes 1B, 2B, 3B, 5D, 6A, and 6D and were named *QGLpd.swust-1BL*, *QGLpd.swust-2BL*, *QGLpd.swust-3BL.1*, *QGLpd.swust-3BL.2*, *QGLpd.swust-5DL*, *QGLpd.swust-6AL*, *QGLpd.swust-6DL.1*, and *QGLpd.swust-6DL.2*. *QGLpd.swust-1BL* was located between *IWB19001* and *IWB70635* and was mapped to the 415,215,474-bp to 565,932,179-bp region of the CS reference genome on chromosome 1BL, exhibiting phenotypic variance explained (PVE) ranging from 13.5% to 14.9% and an average LOD score of 3.7. *QGLpd.swust-2BL* was located between *IWB40455* and *IWB61115* and was mapped to the 697,550,620-bp to 775,179,574-bp region of the CS reference genome on chromosome 2BL, exhibiting PVE ranging from 11.7% to 18.1% and an average LOD score of 4.0. *QGLpd.swust-3BL.1* was located between *IWB41833* and *IWA2177* and was mapped to the 35,322,045-bp to 35,311,024-bp region of the CS reference genome on chromosome 3BL, exhibiting PVE ranging from 9.9% to 16.0% and an average LOD score of 3.8. *QGLpd.swust-3BL.2* was located between *IWB6177* and *IWA6997* and was mapped to the 610,751,868-bp to 633,854,986-bp region of the CS reference genome on chromosome 3B, exhibiting PVE ranging from 12.1% to 17.6% and an average LOD score of 3.9. *QGLpd.swust-5DL* was located between *IWB45948* and *IWB36098* and was mapped to the 464,068,639-bp to 465,006,570-bp region of the CS reference genome on chromosome 5D, exhibiting PVE ranging from 10.0% to 14.0% and an average LOD score of 3.1. *QGLpd.swust-6AL* was located between *IWB1549* and *IWB33868* and was mapped to the 198,366,375-bp to 530,045,849-bp region of the CS reference genome on chromosome 6A, exhibiting PVE ranging from 15.2% to 17.6% and an average LOD score of 4.7. *QGLpd.swust-6DL.1* was located between *IWB55825* and *IWB71500* and was mapped to the 415,215,474-bp to 565,932,179-bp region of the CS reference genome on chromosome 6DL, exhibiting PVE ranging from 17.2% to 21.3% and an average LOD score of 5.2. *QGLpd.swust-6DL.2* was located between *IWB63232* and *IWB16720* and was mapped to the 270,050,614-bp to 386,162,658-bp region of the CS reference genome on chromosome 6DL, exhibiting PVE ranging from 9.0% to 14.2% and an average LOD score of 3.2.

### 3.4 QTL for GW

For GW, a total of two QTLs were mapped on chromosomes 3B and 6D, which were designated as *QGWpd.swust-3BS* and *QGWpd.swust-6DL*, and were significant in three environments (MY19, YL20, and MY20) (**Figure 2**). *QGWpd.swust-3BS* was located between *IWB36263* and *IWB42152* and was mapped to the 36,464,898-bp to 37,256,012-bp region of the CS reference genome on chromosome 3B, exhibiting PVE ranging from 11.4% to14.6% and an average LOD score of 3.5. *QGWpd.swust-6DL* was located between *IWB42114* and *IWB55265* and was mapped to the 469,969,003-bp to 470,043,024-bp region of the CS reference genome on chromosome 6DL, exhibiting PVE ranging from 10.8% to 15.2% and an average LOD score of 3.3.

### 3.5 QTL for LWR

There were two QTLs for LWR on chromosome 7A, and these were designated as *QLWRpd.swust-7AS.1* and *QLWRpd.swust-7AL.2* (**Figure 2**). *QLWRpd.swust-7AS.1* was identified in two environments (YL19 and YL20) and was located between *IWB32677* and *IWB65707*. It was mapped to the 206,709,937-bp to 221,680,636-bp region of the CS reference genome on chromosome 7A, exhibiting PVE ranging from 10.9% to 11.6% and an average LOD score of 13.6. *QLWRpd.swust-7AL.2* was identified in two environments (MY20 and YL20) and was located between *IWB65707* and *IWB6761*. It was mapped to the 221,680,636-bp to 230,296,776-bp region of the CS reference genome on chromosome 7AS, exhibiting PVE ranging from 11.2% to 11.6% and an average LOD score of 2.9.

### 3.6 QTL for TGW

One QTL associated with TGW was detected on chromosome 6B, which was detected in two environments (MY19 and MY20) (**Figure 2**). *QTGWpd.swust-6BS* was located between *IWB55849* and *IWB23139* and was mapped to the 17,229,691-bp to 32,324,802-bp region of the CS reference genome on chromosome 6BS, exhibiting PVE ranging from 14.1% to 16.2% and an average LOD score of 4.2.

### 3.7 QTL for SL

One QTL was identified for SL and was detected in three environments (MY19, YL19, and YL20) (**Figure 2**). *QSLpd.swust-3AL* was located between *IWB34554* and *IWB68722* and was mapped to the 732,779,663-bp to 732,779,682-bp region of the CS reference genome on chromosome 3AL, exhibiting PVE ranging from 16.1% to 17.6% and an average LOD score of 4.5.

### 3.8 QTL for GNS

One QTL was identified for GNS from MX169 and was detected in two environments (YL19 and YL20). *QGNSmx.swust-6DS* was located between *IWB34573* and *IWA5508* and was mapped to the 9,470,596-bp to 13,600,725-bp region of the CS reference genome on chromosome 6DS, exhibiting PVE ranging from 11.4% to 13.8% and an average LOD score of 2.9. The negative additive effect of *QGNSmx.swust-6DS* suggests its involvement in the reduction of the GNS (**Table 2**).

### 3.9 QTL for PH

Five QTLs for PH were identified on chromosomes 2D, 3A, 5A, 6B, and 7B (**Figure 2**). The negative additive effect of these five QTLs suggests their involvement in the reduction of PH. *QPHpd.swust-2DL* was identified in two environments (YL19 and YL20) and was located between *IWB42352* and *IWB3442*. It was mapped to the 570,959,528-bp to 572,786,332-bp region of the CS reference genome on chromosome 2DL, exhibiting PVE ranging from 14.8% to 17.4% and an average LOD score of 4.1. *QPHpd.swust-3AL* was identified in three environments (YL19, MY20, and YL20) and was located between *IWB20268* and *IWB52753*. It was mapped to the 602,919,931-bp to 605,845,913-bp region of the CS reference genome on chromosome 3AL, exhibiting PVE ranging from 15.2% to 19.3% and an average LOD score of 4.3. *QPHpd.swust-5AL* was identified in two environments (YL19 and YL20) and was located between *IWB24934* and *IWB4800*. It was mapped to the 381,761,549-bp to 382,778,448-bp region of the CS reference genome on chromosome 5AL, exhibiting PVE ranging from 11.3% to 17.2% and an average LOD score of 3.8. *QPHpd.swust-6BL* was identified in three environments (YL19, MY20, and YL20) and was located between *IWB6985* and *IWB13533*. It was mapped to the 53,636,781-bp to 715,704,861-bp region of the CS reference genome on chromosome 3AL, exhibiting PVE ranging from 13.4% to 16.9% and an average LOD score of 4.1. *QPHpd.swust-7BS* was identified in two environments (YL19 and YL20) and was located between *IWB44542* and *IWA3507*. It was mapped to the 48,769,162-bp to 58,250,485-bp region of the CS reference genome on chromosome 7BS, exhibiting PVE ranging from 14.1% to 15.0% and an average LOD score of 3.8. The negative additive effects of these QTLs indicate that the identified loci are involved in the reduction of wheat plant height (**Table 2**).

### 3.10 Correlation analysis among characteristics

The GL exhibited significant positive correlations with GW, LWR, SL, and TGW, with correlation coefficients of 0.43 (*p* ≤ 0.001), 0.29 (*p* ≤ 0.01), 0.45 (*p* ≤ 0.001), and 0.43 (*p* ≤ 0.001), respectively. These findings suggest that an increase in GL is advantageous for the enhancement of multiple traits. LWR was highly positively correlated with GL (*r* = 0.29, *p* ≤ 0.01) and SL (*r* = 0.30, *p* ≤ 0.001), but negatively correlated with GW (*r* = −0.71, *p* ≤ 0.001). Moreover, PH displayed negative correlations with GL, LWR, and SL, with correlation coefficients of −0.19 (*p* ≤ 0.05), −0.31 (*p* ≤ 0.001), and −0.20 (*p* ≤ 0.05). Furthermore, GNS was positively correlated with GL (*r* = 0.23, *p* ≤ 0.05) and SL (*r* = 0.67, *p* ≤ 0.001), suggesting that a longer GL and SL would have a lot more GNS. In addition, TGW was highly positively correlated with GL (*r* = 0.43, *p* ≤ 0.001) and GW (*r* = 0.82, *p* ≤ 0.001), but was negatively correlated with LWR (*r* = −0.56, *p* ≤ 0.001), suggesting that TGW, GL, GW, and LWR have at least some gene(s) in common (**Table 3**).

**Table 3:** Correlation analysis of grain length (GL), grain width (GW), grain length-to-width ratio (LWR), thousand grain weight (TGW), spike length (SL), grain number per spike (GNS), and plant height (PH) based on the mean performance of the Mingxian 169 × Pindong 34 recombinant inbred line population in the four environments (MY19, YL19, MY20, and YL20).

	GL	GW	LWR	PH	GNS	SL	TGW
GL	1.00						
GW	0.43***	1.00					
LWR	0.29**	−0.71***	1.00				
PH	−0.19*	0.16	−0.31***	1.00			
GNS	0.23*	0.11	0.06	−0.13	1.00		
SL	0.45***	0.06	0.30***	−0.20*	0.67***	1.00	
TGW	0.43***	0.82***	−0.56***	0.15	0.07	−0.01	1.00

*p = 0.05; **p = 0.01; ***p = 0.001.

## 4 Discussion

Enhancement of the agronomic traits is crucial for increasing cereal yield. The identification and validation of the QTLs associated with traits such as PH, GL, GW, TGW, GNS, SL, and LWR significantly contributes to yield improvement. However, direct selection of these complex quantitative traits presents challenges for breeders. Genomic breeding and MAS represent indirect approaches with high-density marker coverage essential for localizing the QTLs within the wheat genome. Using 90K SNP-Chip genotyping and an RIL consisting of 119 lines, this study identified 20 QTLs associated with agronomic traits across 18 regions on 12 chromosomes.

### 4.1 QTL for GL

In this study, we identified eight QTLs for GL on chromosomes 1B, 1D, 2B, 3B, 4A, 5D, 6A, and 6D. Each of these QTLs originated exclusively from the parent PD34. Collectively, these QTLs explained 9.1%–18.1% of the phenotypic variation. *QGLpd.swust-1BL* was located between *IWB19001* and *IWB70635* and was mapped to the 415,215,474-bp to 565,932,179-bp region of the CS reference genome on chromosome 1BL. *QKlen.caas.1B.2* (Xiao et al., 2011) is a QTL for GL that was linked to marker *Xbarc61* (557,116,000–557,116,146 bp). The physical location of this marker is in proximity to *QGLpd.swust-1BL*, which we have localized, warranting further discussion and exploration. *QGLpd.swust-2BL* was located between *IWB40455* and *IWB61115* and was mapped to the 697,550,620-bp to 775,179,574-bp region of the CS reference genome on chromosome 2BL. *Qkl.ncl.2B.1* (Ramya et al., 2010) was linked with markers *Xbarc159* and *Xwmc317* (644,019,331–783,892,175 bp). Based on the physical location of *QGLpd.swust-2BL* and *Qkl.ncl.2B.1*, they might be the same QTLs for GL*. QGLpd.swust-3BL.1* was located between *IWB41833* and *IWA2177* and was mapped to the 35,322,045-bp to 35,311,024-bp region of the CS reference genome on chromosome 3BL. *QGLpd.swust-3BL.2* was located between *IWB11953* and *IWA6997* and was mapped to the 610,751,868-bp to 633,854,986-bp region of the CS reference genome on chromosome 3B. One QTL for GL was located between *Tdurum_contig6693_787* and *BS00094710_51* (273,250,587–551,894,132 bp) (Russo et al., 2014). Based on the linked markers, *QGLpd.swust-3BL.1* and *QGLpd.swust-3BL.2* might be the new QTLs for GL. *QGLpd.swust-5DL* was located between *IWB45948* and *IWB36098* and was mapped to the 464,068,639-bp to 465,006,570-bp region of the CS reference genome on chromosome 5D. One QTL for GL, *QKl.caas.5DL* (Li et al., 2018), was linked to the marker *IWB65830* (411,170,535 bp) due to the physical location of this marker being close to *QGLpd.swust-5D*, which we have localized. They might be the same QTLs for GL. *QGLpd.swust-6AL* was located between *IWB1549* and *IWB33868* and was mapped to the 198,366,375-bp to 530,045,849-bp region of the CS reference genome on chromosome 6A. The closest marker of *QGl.cau.6A.2* (Zhai et al., 2018) was *TA005615-0600*, and the physical location of *TA005615-0600* was 536,437,136–536,437,190 bp. *QGLpd.swust-6AL* overlapped with *QGl.cau.6A.2*, and further studies are needed to confirm their relationship. *QGLpd.swust-6DL.1* was located between *IWB55825* and *IWB71500* and was mapped to the 415,215,474-bp to 565,932,179-bp region of the CS reference genome on chromosome 6D. A previous study located a QTL for GL that was located between *wPT-3127* and *Xcfd45* (459,178,608–460,317,289 bp) (Williams and Sorrells, 2014). *QGLpd.swust-6DL.1* overlapped with the QTL located by Williams and Sorrells (2014), but further studies are needed to determine whether they are the same or are different QTLs. *QGLpd.swust-6DL.2* was located between *IWB63232* and *IWB16720* and was mapped to the 270,050,614-bp to 386,162,658-bp region of the CS reference genome on chromosome 6D. The QTL *QKl.caas.6DL* (Li et al., 2018) was flanked by *IWA619* (383,747,905 bp), the physical location of which is within the physical interval of the *QGLpd.swust-6DL.2* locus. Consequently, it is imperative to engage in further discussions concerning the potential correlation between them. In conclusion, *QGLpd.swust-3BL.1* and *QGLpd.swust-3BL.2* might represent new QTLs for GL.

### 4.2 QTL for GW

Two QTLs were associated with GW on chromosomes 3B and 6D, explaining phenotypic variation rates ranging from 10.8% to 15.2%. The favorable alleles were all contributed by PD34. As no previous reports exist regarding the QTLs associated with GW on chromosome 3BS, it is plausible that *QGWpd.swust-3BS* represents a novel QTL for GW. The wheat chromosome 6D harbors the genes *qKW-6D* (Chen et al., 2019) and *Q47* (Williams and Sorrells, 2014) for GW. *qKW-6D* was identified on chromosome 6D, showing close linkage to *Xbarc96* (418,280,403 bp). *Q47* was linked with the markers *wpt-3127* and *Xcfd45* (459,178,608–460,317,289 bp). These results suggest that the major QTL for GW identified in this study on chromosome 6D could be an ortholog of *qKW-6D* and *Q47*. More detailed studies focusing on this region need to be conducted to establish their relationship.

### 4.3 QTL for LWR

The QTL for LWR has been rarely reported in wheat. In this study, the LWR QTLs *QLWRpd.swust-7AS.1* and *QLWRpd.swust-7AS.2* were identified in two environments. Due to the diversity of the molecular markers, it was difficult to align and compare the QTLs detected by these studies. *QLWRpd.swust-7AS.1* and *QLWRpd.swust-7AS.2* might represent new QTLs for LWR.

### 4.4 QTL for TGW

The TGW of wheat is an important factor affecting its yield, which is mainly limited by its genetic factors and directly affected by the external living environment (Yao et al., 2019). We detected a QTL for TGW that was present in two environments. *QTGWpd.swust-6BS* was located between *IWB55849* and *IWB23139* and was mapped to the 17,229,691-bp to 32,324,802-bp region of the CS reference genome on chromosome 6BS. Elouafi and Nachit (2004) detected a QTL for TGW linked with the marker *Xgwm518* (88,998,702–88,998,836 bp) around the centromeric region of chromosome 6B (Elouafi and Nachit, 2004). *QTgw.wa.6BS.e2* (Wang et al., 2010) was linked with the marker *Xcfd190* (90,117,178 bp). Assanga et al. (2017) also detected a QTL (i.e., QTL 15) for TGW that was associated with the marker *IWB8809* (159,367,925 bp) (Assanga et al., 2017). Based on the physical location of the previously reported QTL for TGW, *QTGWpd.swust-6BS* might represent a new QTL for TGW.

### 4.5 QTL for SL

In this study, we detected one QTL for SL, *QSLpd.swust-3AL*, which was identified in three environments and was mapped to the 732,779,663-bp to 732,779,682-bp region of chromosome 3A. Previous studies have successfully mapped several QTLs associated with the regulation of GW, GNS, and other agronomic traits on chromosome 3A (Huang et al., 2004; Bennett et al., 2012; Jia et al., 2013; Gao et al., 2015). The majority of these QTLs are primarily located within the centromeric region (approximately 100–450 Mb), the short arm’s distal end (<25 Mb), or the long arm’s distal end (>625 Mb). Only a limited number of QTLs associated with SL have been mapped on chromosome 3A. For example, Yang et al. (2012) detected a QTL for SL, *Qsl3A-4*, on chromosome 3A between *Xbarc356* and *Xbarc314* (172,835,094–712,489,387 bp) that was derived from *Triticum polonicum* L (Yang et al., 2012). Based on the linked markers and its origin, *QSLpd.swust-3AL* is unlikely to be *Qsl3A-4*. In conclusion, *QSLpd.swust-3AL* might represent a novel QTL for SL.

### 4.6 QTL for GNS

We identified one QTL for GNS, *QGNSmx.swust-6DS*, derived from MX169 and flanked by the markers *IWB34573* and *IWA5508* corresponding to the 9,470,596-bp to 13,600,725-bp region of the CS reference chromosome 6DS, which exhibited PVE ranging from 11.4% to 13.8%. A minor QTL for GNS mapped on chromosome 6DS, *QGne.nfcri-6D*, was flanked by *xcfd42* and *xgdm141* and derived from Yu8679, but the physical position of its markers is unknown (Wang et al., 2009). A QTL detected on chromosome 6D for GNS was flanked by *tarc0314* and *tarc1355* and derived from the Japanese high-yield variety Kitahonami (Mizuno et al., 2021). However, due to the lack of data on the physical location of the markers for the earlier QTL, it was not possible to infer whether the QTLs identified in this study are located in the same location as the QTLs for GNS identified earlier.

### 4.7 QTL for PH

Specifically, 24 dwarfing genes (*Rht1*–*Rht24*) have been catalogued in wheat. However, only a few genes for reduced stature have been used in wheat breeding as most have shown strongly negative effects on grain yield (Chapman et al., 2006; Zhang et al., 2013). Thus, it is important to explore and utilize the new QTLs regulating PH. In this study, we identified five QTLs for PH on chromosomes 2DL, 3AL, 5AL, 6BL, and 7BS. Each of these QTLs originated exclusively from the parent PD34. Collectively, these QTLs explained 11.3%–19.3% of the phenotypic variation. *QPHpd.swust-2DL* was located between *IWB42352* and *IWB3442* and was mapped to the 570,959,528-bp to 572,786,332-bp region of the CS reference genome on chromosome 2DL, exhibiting PVE ranging from 14.8% to 17.4%. Liao reported the QTL *Qph.cib-2D.3* with the tightly linked markers *AX-110094567* and *AX-109926918* (484,880,806–494,312,828 bp). *QPht.nfcri-2D* (Wang et al., 2009), flanked by *Xcfd168* (580,011,701 bp) and derived from the Chinese winter cultivar Yu8679, is unlikely to be *QPHpd.swust-2DL. QPHpd.swust-3AL* was located between *IWB20268* and *IWB52753* and was mapped to the 602,919,931-bp to 605,845,913-bp region of the CS reference genome on chromosome 3AL, exhibiting PVE ranging from 15.2% to 19.3%. As there are no previous reports on QTLs associated with PH on chromosome 3AL, it is plausible that *QPHpd.swust-3AL* represents a novel QTL for PH. *QPHpd.swust-5AL* was located between *IWB24934* and *IWB4800* and was mapped to the 381,761,549-bp to 382,778,448-bp region of the CS reference genome on chromosome 5AL, exhibiting PVE ranging from 11.3% to 17.2%. *Rht12* (Ellis et al., 2005) was mapped to a single locus that was tightly linked to the microsatellite marker *WMC410*. The physical position of *WMC410* was 678,293,793–678,293,910 bp. *Rht9* (Ellis et al., 2005) was mapped to a single locus that was tightly linked to the marker *BARC151* (558,340,037 bp). *QPH.caas.5AL.1* (Li et al., 2018) was mapped between *IWB11226* and *IWA3827* (476,659,584–524,245,503 bp). The QTL *Qph.sau-MC-5A* for PH was mapped to the 563,833,704-bp to 568,561,734-bp region of the CS reference genome on chromosome 5A (Guo et al., 2013). *QTL Qph.cib.5A.3* was linked with the markers *AX-108925104* and *AX-108975089* (430,602,625–439,190,927 bp) (Liao et al., 2022). Based on its physical position, *QPHpd.swust-5AL* might represent a novel QTL for PH. *QPHpd.swust-6BL* was located between *IWB6985* and *IWB13533* and was mapped to the 53,636,781-bp to 715,704,861-bp region of the CS reference genome on chromosome 3AL, exhibiting PVE ranging from 13.4% to 16.9%. *QPht.dms-6B.2* (Zou et al., 2017) was mapped between *Kukri_c59960_211* and *Ku_c59960_1939*, and its physical position was 570,923,543–641,291,932 bp. *QPH.caas-6BL* (Li et al., 2018) was mapped between *IWB41570* and *IWB73837*, and its physical position was 687,554,758–710,102,372 bp. *QPht.dms-6B.2* and *QPH.caas-6BL* overlapped with *QPHpd.swust-6BL*, but further studies are needed to confirm their relationship and to determine their relationships with the other QTLs on chromosome 6BL. *QPHpd.swust-7BS* was located between *IWB44542* and *IWA3507* and was mapped to the 48,769,162-bp to 58,250,485-bp region of the CS reference genome on chromosome 7BS, exhibiting PVE ranging from 14.1% to 15.0%. *Qph.nau-7B* (Jia et al., 2013) was mapped between *Xwms537* and *Xmag2110*. The physical position of *Xwms537* was 26,803,596–26,803,806 bp. *QHt-7B-1* (Liu et al., 2011) was mapped between *Xcau130* and *Xgwm537*. The physical position of *Xgwm537* was 26,803,596–26,803,806 bp. Based on the physical position, *QPHpd.swust-7BS* might represent a novel QTL for PH. In conclusion, *QPHpd.swust-2DL*, *QPHpd.swust-3AL*, *QPHpd.swust-5AL*, and *QPHpd.swust-7BS* might represent new QTLs for PH.

## Data availability statement

The datasets presented in this study can be found in online repositories. The names of the repository/repositories and accession number(s) can be found in the article/supplementary material.

## Author contributions

LZ: Writing – review & editing, Writing – original draft, Formal analysis. YL: Writing – original draft, Formal analysis. XZ: Writing – original draft, Data curation. GJ: Writing – original draft, Investigation. HC: Writing – original draft. YW: Writing – original draft, Data curation. JZ: Writing – original draft, Formal analysis. CM: Writing – original draft, Formal analysis. XL: Writing – original draft, Investigation. KH: Writing – review & editing, Investigation. SY: Writing – review & editing, Investigation. JW: Writing – review & editing, Formal analysis. DH: Writing – review & editing, Resources. YR: Writing – review & editing, Investigation. LC: Writing – review & editing, Supervision. XLZ: Writing – review & editing, Funding acquisition.
